# On the Importance of Investigating Data Structure in Miniaturized NIR Spectroscopy Measurements of Food: The Case Study of Sugar

**DOI:** 10.3390/foods12030493

**Published:** 2023-01-20

**Authors:** Giulia Gorla, Paolo Taborelli, Cristina Alamprese, Silvia Grassi, Barbara Giussani

**Affiliations:** 1 Department of Science and High Technology, University of Insubria, Via Valleggio 9, 22100 Como, Italy; 2 Department of Food, Environmental and Nutritional Sciences, University of Milan, Via Celoria 2, 20133 Milano, Italy

**Keywords:** food analysis, miniaturized-spectroscopy, multivariate error, near infrared, uncertainty

## Abstract

Alongside the increasing proofs of efficacy of miniaturized NIR instruments in food-related scenarios, it is progressively growing the number of end-users, even incentivized by the low-cost of the sensors. While attention is paid to the analytical protocol–from sampling to data collection, up to the data processing, the importance of error investigation in raw data is generally underestimated. Understanding the sources and the structure of uncertainty related to the raw data improves the quality of measurements and suggests the correct planning of the experiments, as well as helps in chemometric model development. The goal of chemometric modeling is to separate information from noise; therefore, a description of the nature of measurement error structure is necessary. Among the different approaches, we present the study of the Error Covariance Matrices (ECMs) and their decomposition in a bilinear structure as a powerful method to study the main sources of variability when using miniaturized NIR sensors in the actual way of use. Granulated and lump sugar samples were chosen as the case study and analyzed with two miniaturized spectrometers working in the NIR regions around 1350–2550 nm and 900–1750 nm, respectively, in dispersive reflectance mode. Results show that having some insights on multivariate measurement errors associated with spectra could be interesting in paving the way for several applications.

## 1. Introduction

In recent years, increasing proofs of the efficacy of miniaturized NIR instruments [1] for various applications [2] in food-related scenarios are spreading over the literature. Alongside it and incentivized by the low cost of the sensors and the easy-to-use interfaces provided by the manufacturers, the number of end-users who could use guidance in choosing the acquisition strategies that would better fit their needs is progressively growing [3]. Data collected with portable NIR sensors are often subject to multivariate data analysis. While more effort is made in sampling properly and optimizing the analytical procedure, including the final data treatment, fewer studies are directed to the analytical accuracy and reliability of various miniaturized spectrometers available in the market [4]. However, there is an even more overlooked aspect: the nature of the measurement errors, thus the error characterizing the raw data. Multivariate data treatment aims to separate information from noise: a description of the structure of errors taking the measurement errors into account is thus necessary [5]. Understanding the sources and structure of uncertainty in raw data could improve the quality of measurements by helping to find the actual causes of the error itself [5,6]. In this context, the characterization of the spectroscopic errors and the identification of the sources of variability that affect them could allow the extraction of chemical information [7,8,9,10] (as it is already confirmed for chemical processes with the estimation of measurement uncertainty [11,12]). Moreover, it could lead to the development of ad hoc signal preprocessing [13,14] or to the better application of the existing ones [15]. Uncertainty is a permanent part of every measurement from different instruments and methods [16], and the best thing to do with it is to ensure its monitoring and minimization [6]. Indeed, as the development of quantitative linear models in analytical calibration became ubiquitous, the knowledge of measurement uncertainty in experimental data analysis showed up as needed for the diagnostics of models (e.g., the figure of merit [17]).

Nevertheless, the high importance of the uncertainty of the measurements is to be highlighted in the monitoring of measurement systems, and it could provide several helpful pieces of information about the experimental and instrumental conditions. The estimation of spectrometers’ recalibration times and the evaluation of model maintenance (i.e., the calibration transfer strategies between different instruments and within the same devices) [18,19,20,21,22] could undoubtedly benefit from the identification and characterization of the sources of variance related to the analysis in different conditions. As a matter of fact, if one would intend the instrument life as a process, and every session of analysis as a steady state of the process, knowing the uncertainties at each stage represents a fundamental notion of taking them under control [23,24,25,26]. The identification of the reliable shape of the error could undoubtedly help in monitoring the instrumental performances. Indeed, if adequate care of the outliers is performed during the error estimation, the error of new spectra could be considered as tolerable or not, paving the way for the monitoring and maintenance of sensors. 

This study has a twofold purpose. On one hand, it aims to understand and characterize the uncertainty features related to the use in real conditions of two handheld miniaturized NIR instruments working with different configurations and in different spectroscopic ranges. On the other hand, it intends to propose a practical and feasible methodology to investigate raw data uncertainties, usable even by those who are not experts in data analysis. The scope of this work is to give an intuitive method to apply whose results are easy to interpret through images and not numbers, which is generally what multivariate techniques (wildly used explorative multivariate techniques) aim for.

While it is known and already studied that NIR reflectance spectra are dominated by constant and multiplicative offset noise that have a strong interaction, which occurs for benchtop and portable sensor signals [27], other sources of correlated noise are not so investigated. For example, the influence of the timing between the background collection or performing the measurements in different analytical sessions. This work also sheds light on these contributions and offers a methodology to investigate their effects. 

For this purpose, external dispersive reflectance spectra of sugar (sucrose) in different forms (lump and granulated sugar) were acquired in replicates with two miniaturized spectrometers working with different spectral ranges and optical configurations. This sample has been chosen due to its well-known composition and behavior and the possibility of having different commercially available packaging, besides the food industry interest, particularly in the confectionery field. The dominant structure in the variability of replicates and the corresponding factors were studied through preliminary exploratory analysis with Principal Component Analysis (PCA). Then, the method proposed to check the measurement errors is the study of the Error Covariance Matrices (ECMs), estimated by the experimental approach.

## 2. Materials and Methods

### 2.1. Samples and Spectrometers

Granulated and lump sugar was purchased from a local supermarket and maintained at room temperature in a protected environment for the duration of the experiments. 

Near-Infrared spectra were acquired by using two miniaturized spectrometers. The parameters reported below were used and are related to what producers declare.

- AvaSpec-Mini-NIR (Avantes, Apeldoorn, NL, The Netherlands) with a reflection fiber probe (7 × 400 µm fiber, 2 m length, SMA term), with an integration time of 15 ms and the average scan set at 10. For each spectrum, 236 data points were obtained in the range 972–1701 nm.
- NeoSpectra Scanner spectrometer (Si-Ware Systems, Menlo Park, CA, USA), at a time scan of 5 s without data interpolation. 74 data points for each spectrum were acquired in the range 1351–2559 nm. Data were acquired with direct contact analysis.

It is worth noting that there is no harmonization in providing descriptions of instrumental parameters and every manufacturer has their own way of doing it.

The possible influence on raw data measurement error due to the time elapsed between background recording and the analytical session was investigated. Fifteen analytical replicates consisting in sample repositioning over the reading window were collected for each sample during six independent analytical sessions. Concerning the background, two cases were investigated: spectral background acquired before the analysis of each sample (thus every 15 replicates of the same sample) or at the beginning of each session of analysis. In the last case, background was recorded, other spectra (not reported here) were acquired to simulate the use of the spectrometer in an ordinary research facility for control analysis for approximately 20 min, and then sugar samples spectra were recorded. 

For each of the two background conditions, three independent sessions of analysis were performed. A total of 90 experimental replicates with each instrument was acquired.

A scheme of the resulting dataset for each experiment is in Supplementary Figure S1. Spectra were acquired with the providers’ app (NS-Scanner v.1.0.2) and software (AvaSoft Version 8.12) and imported in Matlab R2021a (Mathworks) for further elaborations. 

### 2.2. Methods

Mean absolute and relative standard deviation calculated as the mean over the spectra of the standard deviation and relative standard deviation calculated for each wavelength were evaluated as the first step to study the raw data. RMS statistics [28] usually studied for reproducibility and the mean Signal to Noise ratio [29] was also calculated. The RMS statistic is defined as the averaged root mean square of differences between the different subsamples scanned at n wavelengths. The Signal to Noise ratio is defined as the ratio between the average spectrum and the absolute standard deviation [30].

Exploratory data analysis was conducted to study the main information contained in the spectra and to manage outlier detection. With these purposes, and to allow the identification of trends and patterns in the data, related to known variabilities, Principal Component Analysis (PCA) was used. 

Multivariate measurement error was calculated and investigated as proposed by Leger et al. [27]. As the first step, the error matrix was obtained as the difference between each replicated spectrum and the mean of the replicates (considered the real value). Subsequently, the variance-covariance (**Σ**–it is also called covariance matrix) and correlation matrices were calculated and studied. 

The error covariance matrix describes the correlation between the errors at the different wavelengths. The diagonal of the matrix gives an idea about the errors’ uniformity in the spectra (homoscedastic), while non-uniform values indicate that errors are not constant along the spectra (heteroscedastic). The off-diagonal elements give information on the covariance of the measurement errors. Where the covariance matrix gives the magnitude of the relationship among the errors, the correlation matrix, derived from the covariance matrix, indicates the underlying structure of this relationship, giving a piece of complementary information [5]. Even if these matrices could discourage final users from employing this mathematical tool, the calculations are quite simple in any programming environment, and the interpretation of their results is graphical and thus easy-to-see. 

Since errors are often dominated by a bilinear structure, PCA can be used to deduce the main structure by allowing simple interpretation related to the magnitude of the independent error components. Each covariance matrix was so decomposed by bilinear modeling through PCA to identify the main qualitative trends and to compare them at the experimental level. PCA is proposed for this application, although there are sometimes better methods [31], because it is at the root of many chemometric approaches, well-known, feasible, and applicable by many scientists. Moreover, it has proved effective in investigating NIR spectroscopic measurement errors [27]. PCA decomposition can provide a simplified representation of the error covariance matrix. The number of factors required to reconstruct the covariance matrix and their shape can help interpret the underlying errors [32]. 

The cumulative variance for the PCA on the error covariance matrix was compared with the cumulative percentage variance for the original residual matrix (**Ê**) to assess the contribution of uncorrelated errors. If results are similar the uncorrelated error is low, the contrary is true if the differences are consistent. The original residual matrix (**Ê**) is calculated by subtracting to each spectrum the mean of all spectra. The trends on the diagonal of the covariance matrices and the correlations were explored and then resumed as a K index [33]. This index is a redundancy index that could be used to resume the correlation of a set of multivariate data. It ranges between 0 and 1 by assuming the lowest value when all the variables are uncorrelated and the highest when they are correlated. The imbedded correlation is the minimum correlation within the data and in here it is used to quantify the correlation within the error matrices for different instruments. 

Data elaboration and analysis were performed with PLS toolbox 8.9.1 (Eigenvector Inc., Manson, WA, USA) and in-house routines programmed in MATLAB 9.10.0.1602886-R2021a (Mathworks Inc., Natick, MA, USA). The source code is available upon request [34].

## 3. Results and Discussion

### 3.1. Spectra

NIR spectral range (800–2500 nm) could be divided into three regions, whose borders are not severely strict but at least useful for generalization. Bands resulting from electronic transitions, higher-order overtones, and other sorts of combination modes arise in Region I (800–1200 nm). Then, the first and second overtones of the XH (X = C, O, N) stretching vibrations and various types of combination modes appear in Region II (1200–1800 nm). Finally, Region III contains mainly bands attributed to the combination modes except for the second overtone of the C=O stretching vibrational mode [35]. As a general consideration, miniaturized spectrometers do not cover all the range of three NIR spectral regions. AvaSpec-Mini-NIR covers a small part of Region I and almost the entire Region II. NeoSpectra Scanner covers a part of Region II and Region III.

**Figure 1:**
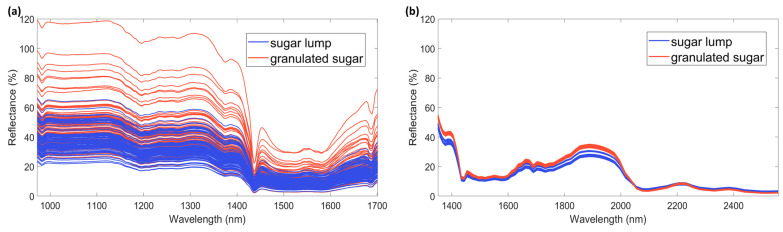
Spectra of sugar samples acquired with the miniaturized NIR spectrometers (90 acquisition with each instrument). (**a**) AvaSpec-Mini-NIR; (**b**) NeoSpectra Scanner.

Spectra acquired in different ranges of spectral radiation and with different instrumental technologies (optical fiber or direct contact window) obtains distinct chemical and physical information as it can be seen from Figure 1, where the raw spectra collected with the two spectrometers used in this study are shown. It is worth knowing that the instruments did not automatically correct the scattering effects for the samples. The reproducibility and quality of spectra of the same samples with different compactness for the two spectrometers are reported in Table 1. The descriptive statistics chosen were used to have a first look into the data, before further investigations. These statistics are limited by the lack of direct recognition of the reasons influencing spectra changes. However, even at a first sight it is clear that the spectra collected by AvaSpec-Mini-NIR are less reproducible for granulated sugar than for lump sugar. This observation was confirmed by the calculation of absolute and relative standard deviation values, as well as RSM statistics and Signal to Noise ratio. 

By investigating the values obtained for NeoSpectra Scanner data, it is possible to identify a lower standard deviation and a higher signal to noise ratio for granulated sugars than for lumps, whereas a higher standard deviation was estimated for the RMS statistic of sugar lumps than granulated sugar. The direct comparison of values reported in Table 1 for the two spectrometers showed how the trends between spectra of the type of sugar samples are inverted and the values obtained for the AvaSpec-Mini-NIR spectra are higher than those of the NeoSpectra. However, it is important to consider even the differences in the absolute reflectance values registered. NeoSpectra signals were found with lower reflection values than AvaSpec-Mini-NIR spectra.

Differences in the instrumental technologies (e.g., use of optical fiber, dimension of the spectral window, light source) strictly influence the path of light and, thus, the obtained signal. The sensors can scan different sample areas depending on the technological features, and this could be a key point when dealing with inhomogeneous samples [35]. Moreover, the two spectrometers cover different wavelength ranges, thus spectral regions are intrinsically characterized by different energies, also correlated to different penetration ability in the samples.

Consistently with what was already pointed out by the literature on this kind of instrumentation [36], a strict dependence on the considered application [2] and the particle size of the sample has to be considered [37,38]. In this sense, other interesting aspects arise from the evaluation of the compactness of the samples, which influences the spectra at the reproducibility level. 

### 3.2. Exploratory Analysis

#### 3.2.1. AvaSpec-Mini-NIR

A PCA model was carried out on raw mean-centered spectra. Outliers’ evaluation was carried out based on the Hotelling’s T^2^-statistic and Q statistics. The former is a generalization of Student’s t-statistic used in multivariate hypothesis testing to assess unusual variation inside the model while the latter is used to identify samples that are not explained by the model of principal components [39]. One granulated sugar spectrum was identified as outlier on both T^2^ and Q. Looking at the contribution plot for both directions (not shown here), intensity values higher than those of the mean samples and, generally, more defined shapes were identified. The sample was then removed from further elaboration. 

**Figure 2:**
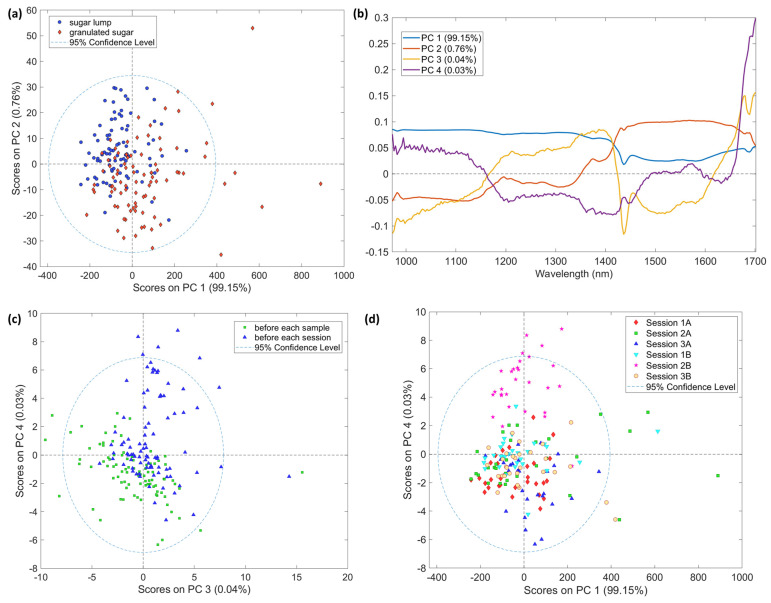
PCA models on AvaSpec−Mini−NIR spectra. (**a**) Score plot PC1 vs. PC colored by samples; (**b**) Line loading plot; (**c**) Score plot PC3 vs. PC4 colored by time of background; (**d**) Score plot PC1 vs. PC4 colored by session and time of background where A and B in the legend stands for background before each sample and before each session, respectively.

Figure 2 shows score and loading plots labeled as a function of different factors. Interestingly, all the samples regardless of their different compactness lied in the 95% ellipse of the score plot. The tendency emerging from the labeling based on the type of sample in the PC1 and PC2 space, explaining 99.15 % and 0.76 % of the data variance, respectively, manifests that the samples outside the ellipse are all granulated sugars. However, it is worth noting that the percentage of outside data remains below 5% of the total spectra analyzed. Regarding the other factors studied in the analysis (background timing and session of analysis), there are no identifiable patterns in the scores space. The intrinsic variance of the spectra of granulated sugar seems to be higher than that of the sugar lumps for this instrument. A rationale for that could be ascribed to the analytical variability included in the acquisition process: if with the lumps the presentation of the sample to the optical fiber is more easily standardized, this is not true for the granulated sugar. A standardized quantity of sample was presented to the spectrometer by incomplete flattening so that each particle in the sample would present its own surface to the incident light. The results obtained are in accordance with what emerged from previous works with diffuse reflectance NIR spectrometers [37,40]: a strong influence on the spectra reproducibility depending on the packing density and the light paths due to scattering effects emerged.

It is noteworthy how on the score plot of PC3 and PC4 (0.04% and 0.03% respectively) it could be identified a trend related to the timing of background acquisition and when plotting PC1 and PC4 (99.15% and 0.03% respectively) the sessions of analyses result slightly clustered. Even if these interpretations are qualitative and, as expected, the differences between samples are not sufficient to group them according to some secondary factors of acquisition, the distribution of the samples in the PCA scores space could not be seen as random. 

#### 3.2.2. NeoSpectra Scanner

**Figure 3:**
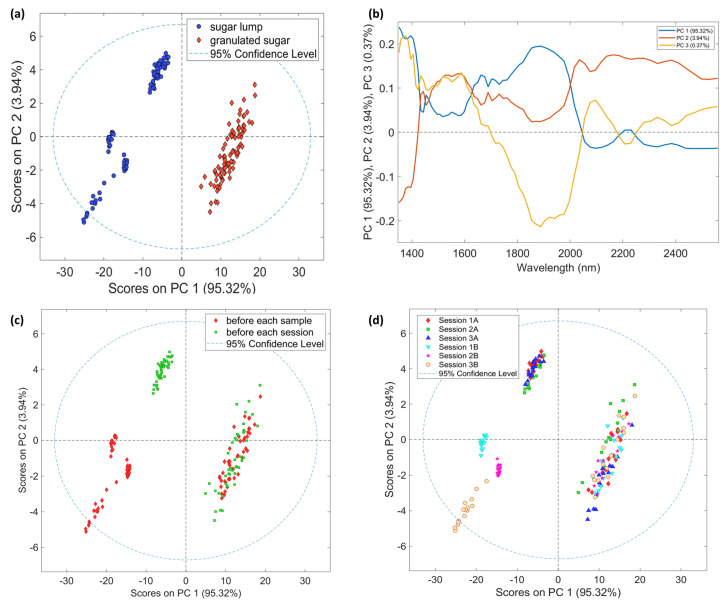
PCA models on NeoSpectra Scanner spectra. (**a**) Score plot PC1 vs. PC2 colored by samples; (**b**) Line loading plot; (**c**) Score plot PC1 vs. PC2 colored by time of background; (**d**) Score plot PC1 vs. PC2 colored by session and time of background where A and B in the legend stands for background before each sample and before each session, respectively.

A PCA was performed on all the sugar spectra by only mean-centering the data. In this case, the main variance in the spectra represented in the first PC of the scores is related to the sugar form. The score and loading plots in Figure 3 show clear grouping on PC1 and PC2 (95.32 and 3.94 % of variance, respectively) related to the compactness of the sugar. As it could be emphasized from labeling and coloring based on background timing and acquisition sessions, the distribution of sugar lump spectra could not be seen as unsystematic for these factors. Besides the non-random distribution, it is not trivial to find the reason in the loading plot. Indeed, loadings shapes are rather complex and they represent several scattering contributions [35]: as, e.g., light path lengths, pack density, and incidence angle.

The differences between this sensor and the previous one signals and PCA models could be explained by the different spectral range, the acquisition window dimensions and the technical configuration of the instruments. Of course, not only the range is an important factor, but also the acquisition system that for NeoSpectra consists of a window with a diameter of 2 cm that covers a larger sample surface than the optical fiber. Moreover, the acquisition with NeoSpectra assumed that the sample is close to the light source and the detector. 

### 3.3. Multivariate Measurement Error

Multivariate measurement errors were investigated for the spectroscopic data, as common, describing them by means of the error covariance matrices (**Σ**). The errors were estimated by experimental replicates. **Σ** is one of the most complete ways to characterize errors in a vectorial measurement (as a spectrum can be intended). It is a symmetric matrix whose size is the number of data points measured by the sensor and it is scale-dependent, which means that differences in the scales of values are related to differences in the dimension of the error of original data. As previously mentioned, the diagonal elements represent the error variance for each wavelength and the off-diagonal values represent the covariance between wavelengths. In addition, it is possible to derive the correlation matrix whose interpretation allows to obtain a scale-independent panoramic of the correlation between channels of signal acquisition.

In this context, it is worth noting the emphasis needed on the definitions of replicates and sample since depending on that different sources of variation should be considered in the evaluation of the measurement error. According to the preliminary exploratory investigation, **Σ**s of the instruments were calculated considering the sugar forms as two distinct samples for both sensors. As the “replicate”, the replacement replicate [5] as intended in the literature was considered. When referring to “session replicates” the same sample acquired during a single session of analysis is considered (15 replicates for each session). Moreover, “background replicates” are to be intended as some session replicates (three groups of session replicates for each sample) acquired under the same condition of background (before each sample or at the begging of an analytical session). Covariance and correlation matrices obtained for each type of replicates were calculated using the same number of replicates and then compared. 

#### 3.3.1. AvaSpec−Mini−NIR

**Figure 4:**
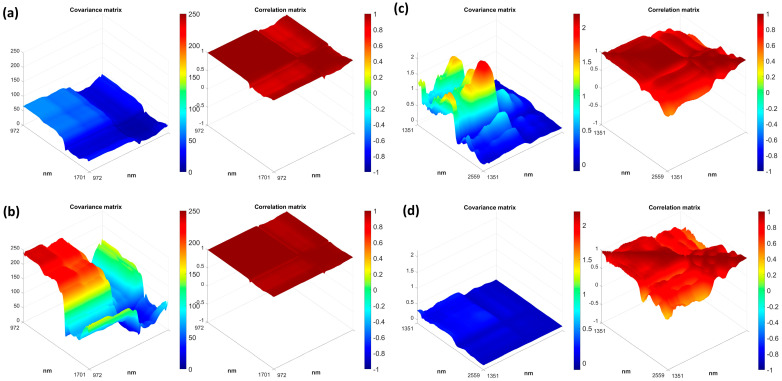
Measurement error covariance and correlation matrix for sugar lump spectra (**a**) and granulated sugar spectra (**b**) acquired with AvaSpec−Mini−NIR and for sugar lump spectra (**c**) and granulated sugar spectra(**d**) acquired with NeoSpectra Scanner.

The interpretation of error covariance matrices allows the identification of the type of noises and thus of the sources of errors. Additionally, the identification of the error variance related to the different elements of a measurement vector (wavelength of spectra) is permitted according to the heteroscedasticity typical for chemical measurement vectors. To consider the comprehensive error that occurred during the experimentation, all the sources of variances introduced by session replicates and/or background replicates were incorporated by performing the calculation on the overall dataset divided on sample type basis. Figure 4 shows the measurement error covariance and the correlation matrices for the two sugar samples under investigation. As expected, and besides the univocal chemical characteristics, the different packaging form of the samples has an influence on the deviations of the signal from its real value and so, on its error. This was in congruence with what was obtained from the preliminary signal studies regarding the standard deviations and RMS statistic of the signals.

From a visual inspection of Figure 4a,b some preliminary considerations about the noise structure could arise. Similar structures could be identified for the two samples. Offset noise and multiplicative noise could be qualitatively identified in the calculated matrices in agreement with what was found in the literature. These types of errors are inferred to be the main ones responsible for the typical measurement error in the NIR region [15,27] and are showed by the fact that the value of the error is all above 0 and it is proportional to the wavelength, respectively. Shot noise could also be identified, as found in another study involving this kind of instruments in powder samples analysis [15]. A possible reason for the presence of this kind of error is related to the extensive electronic component involved in generating the signal in the instruments. The main effect could be inferred as the one related to the offset noise due to the almost flat correlation matrix. Besides that, some regions systematically appear throughout the surface with smaller levels of correlation proving the influences of other sources of error. 

The inspection of the error covariance matrices for the two samples showed an interesting difference in the absolute value of the errors even if the general pattern is almost the same. This could be interpreted by looking at the different reproducibility of the light path for the two samples ascribable to their intrinsic physico-chemical characteristics. 

The presence and the type of errors were validated by applying PCA for decomposing **Σ** and by comparing the structures and shapes in the loading plots (Supplementary Figure S2) which are similar, at least for the first two PCs.

The following step was the calculation of the residuals matrix (**Ê**) that was obtained by subtracting at each spectrum the mean of all the spectra in the dataset. Subsequently, a PCA model was carried out with the aim of identifying significative components and compare the variance values with those of the PCA on the error matrix. This could achieve an idea of the magnitude of the independent errors. Three components could be chosen for interpretation by joining the information of log(eigenvector) curve and cumulative variance. The cumulative variance for the PCA performed on the error covariance matrix is reported in Table 2, together with the cumulative percentage variance for the original residuals matrix (**Ê**). The small differences between these values for the same samples indicate that the error variance has a small contribution due to independent noise terms. Moreover, the uncorrelated errors, when comparing the two samples, could be intended as very similar in magnitude. As a results, it could be possible to state that spectra recorded for the two sugar samples have different absolute intrinsic errors when using this sensor and that the error is, mainly, but not only, related to the scattering conditions. 

The error variance–covariance and the correlation matrices for the granulated sugar were also calculated by including the outlier identified during the exploratory analysis (not shown here). The shape of the overall structure did not change qualitatively, whereas the absolute value of the covariance matrix resulted as higher as those without the outlier sample. The errors previously described showed stable values when removing other random replicates.

##### Study of the Influences of Time of Background and Session

**Figure 5:**
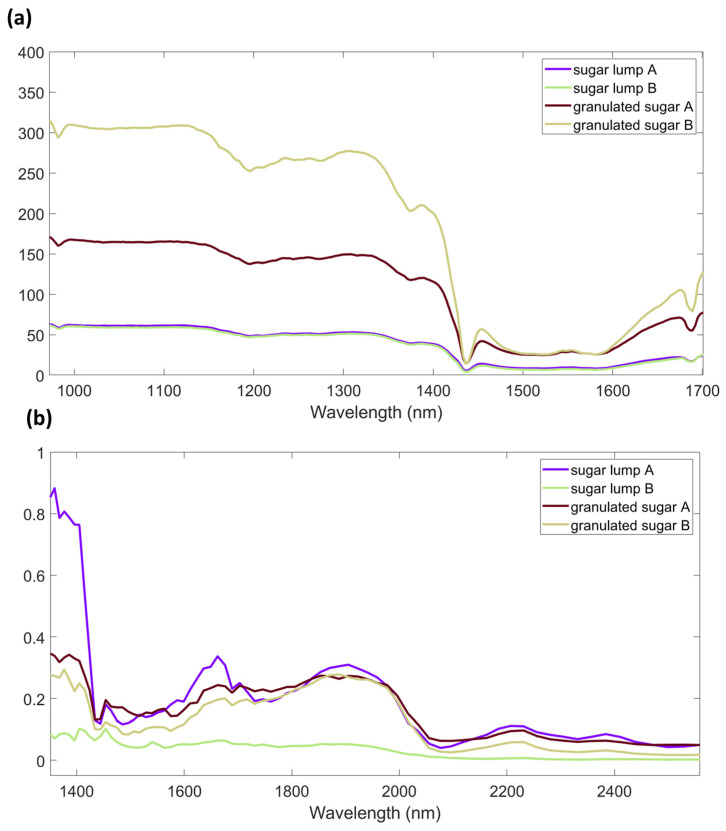
Diagonal of the Covariance Matrices calculated over background replicates spectra collect with (**a**) AvaSpec−Mini−NIR and (**b**) NeoSpectra Scanner. A stands for “background acquired before each sample”. B stands for “background acquired before each session”.

The following steps consisted in considering the error of the individual sugar samples according to the background timing and then to the session. Considering the two types of background replicates (A–background acquired before each sample and B–background acquired at the beginning of the session) the inspection of the diagonal of the covariance matrices was performed (Figure 5a). At first glance, curves show similar shape with different magnitude, and they seem similar to the curve obtained using samples in all background and analytical session conditions (Supplementary Figure S3). The diagonal values are not constant over the wavelengths, and they vary in accordance with the magnitude of the spectra, indicating non-homoscedastic noise patterns that could be linked to the noise structured for shot noise and multiplicative noise. An interesting result appeared to be how the more standardized compactness of sugar lumps obtain qualitatively the same error structure independently from the background timing, whereas this is not true for the granulated sugar. In the latter case, the background acquisition before each sample seems to lead to a lower overall error.

**Figure 6:**
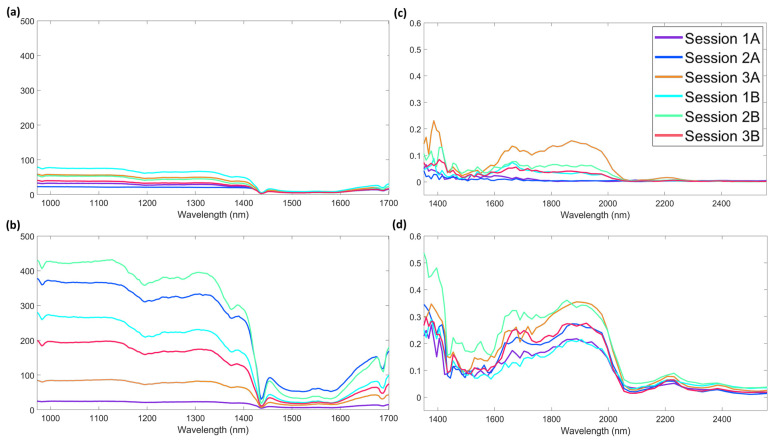
Diagonal of the error covariance matrices calculated over session replicates for AvaSpec-Mini-NIR data: (**a**) sugar lump spectra (**b**) granulated sugar spectra and for NeoSpectra Scanner data: (**c**) sugar lump spectra (**d**) granulated sugar spectra.

The error covariance obtained from the multivariate error of the session replicates is reported in Figure 6a,b, where the diagonals of the matrices are reported. Each session is identified with a number from 1 to 3 and A and B are used to flag the background conditions. Expectedly, as emerged for the background replicates and depending on the sample, the absolute values are different, being lower on average for sugar lumps than for granulated sugars. As for the shapes of the diagonals, the behavior of typical NIR error is always identifiable but the absolute values are different for different sessions within the same sample. No remarkable differences could be observed also with the diagonal obtained using samples belonging to all the sessions. In addition, even if the main contributions could be inferred as the same, more designated shapes are visible whereas the absolute value became bigger. The increasing resolution of the error covariance could not be seen as characteristic of only one spectral region as for the error is not ascribable to specific peaks. In addition, the discrepancy in values among the sessions is bigger when considering granulated sugar than for sugar lumps.

The calculation of the correlation K index on the error data matrix (*n* replicates × *p* wavelength) was used to evaluate the total quantity of correlation contained in a data. An interesting aspect to consider is that in this case the number of *p* is greater than the number of *n,* so the rank of the correlation matrix is *n*. According to Todeschini et al. [33] the minimum correlation within the data (15 replicates × 236 data points) is 0.94. The K value related to the different sessions were calculated. As already emerged from the visual inspection of the correlation matrix, the correlation in these sets of data approaches to 1 (K index of 0.99 and 1 were obtained). Unexpected trends over the sessions are non-identifiable due to the small variations that occur at the third decimal. There is no difference in the K correlation index error for the granulated sugar and the sugar lumps. This help to state that the magnitude of independent error is very small in this kind of data.

#### 3.3.2. NeoSpectra Scanner

The same procedures followed for the AvaSpec-Mini-NIR spectra were applied to the NeoSpectra Scanner data. The errors and the corresponding characterization through covariance and correlation matrices were obtained by dividing the dataset according to the sample type and by using all the replicates for the estimation (90 replicates × sample). 

In Figure 4c,d the measurement error covariance and correlation matrices for the two samples under investigation are reported. As it can be seen, the packaging form of the samples to the spectrometer influenced the error in the measurements in an opposite way compared to the previously commented spectrometer. By visual inspecting Figure 4, some considerations could be achieved about noise. The expected contributions were related to the offset noise, shot noise, and multiplicative noise, and their patterns can be graphically identifiable in both of the calculated matrices. 

The absolute covariance values were lower for granulated sugar spectra than for the sugar lump ones, even if the general shapes are the same. PCA was used to decompose **Σ** and evaluate the presence and the type of errors and to compare the structures and shapes in the loading plots (Supplementary Figure S4). Similar behavior was proven. 

The cumulative variance for the PCA performed on the error covariance matrix is reported in Table 3, together with the cumulative percentage variance for the original residual matrix (**Ê**). The differences between these values for the sugar lump spectra indicate that the error variance has a small contribution due to independent noise terms, bigger than in the case of AvaSpec-Mini-NIR spectra. Observing the values of variance calculated for the granulated sugar it is worth noting that the variance in the residuals is lower (of about 10%) than that of the error covariance matrix. For the latter, the uncorrelated errors could be seen as a more consistent contribution. Spectra acquired with NeoSpectra scanner were identified as part of two groups from the exploratory analysis and the results here reported show how the measure of the samples have even different absolute error associated.

##### Study of the Influences of Time of Background and Session

Measurement errors of the two sugar samples were calculated according to the background timing and then to the sessions. The diagonals of the covariance matrices (Figure 5b) were investigated by considering two types of background replicates (A–background acquired before each sample and B–background acquired at the begging of the session). As expected, the change is in accordance with the magnitude of the spectra, indicating non-homoscedastic noise patterns (as occurred in the curve calculated on the whole data set–Supplementary Figure S3). It is interesting to note how, even if with different absolute values and specific shapes, the overall error structures obtained are not really mismatched with the ones from the other sensor. At shorter wavelengths, there is the maximum percentage of reflectance and the maximum covariance in the error, whereas at longer wavelengths the covariance in the error structure decreases according to the mean spectra. A noticeable characteristic of the error covariance could be that the more standardized geometrical setting between sample and spectrometer, possible for the sugar lumps, does not allow to obtain an error structure independent from the time at which the backgrounds were acquired, whereas this seems the result for granulated sugar. This result highlights how the differences in the spectral range are important when evaluating miniaturized NIR sensors. Another relevant aspect of extreme importance is the size of the spectral window in relation to the heterogeneity or homogeneity of the sample. Indeed, in the case of NeoSpectra Scanner, the presentation to the spectrometer of the sugar lumps did not allow to cover the entire window influencing the error sources in the procedure by showing that the choice of miniaturized instruments must rely also on this aspect. The reproducibility over replicates was obtained by placing the lumps always with the same orientation and alignment to the detector, which is placed in the middle of the lamps. Despite that, the reproducibility of the spectra could have been partially influenced by the laboratory light. 

The error covariance obtained for the multivariate error of the session replicates is reported in Figure 6c,d, where the diagonals of the matrices are shown. Each session is identified with a number and A and B are used to flag the background conditions. Contrary to what can be seen when calculating the error on the overall spectra of each sample, when dividing the replicates according to the sessions, the absolute values are on average lower for sugar lumps than for granulated sugar. These results could suggest that the reason for the overall outcome is the combination of sources of error from different sessions. As for the shapes of the diagonals, even if the general NIR tendency is identifiable for both the samples (and not remarkably different from the one obtained on the whole data set–Supplementary Figure S3), the variance between wavelengths for some sessions of sugar lump spectra results low on every channel of acquisition. That is the case for Session 1A and 2A whose noise contributions could be inferred as a low effect of offset noise and uncorrelated error.

The calculation of the correlation K index on the error data matrix (*n* replicates × *p* wavelength) was used to evaluate the total quantity of correlation contained in the data. The minimum imbedded correlation within the data acquired within each session (15 replicates × 74 data points) is 0.81.

The K value related to the different sessions were calculated and are reported in Table 4. The visual inspection of the error correlation matrices evidences the contribution of more than only offset noise for both the samples. No evident trends over the session are identifiable. K correlation index errors for granulated sugar were found on average bigger than those of sugar lumps indicating that for the sugar lump spectra the error in the correlation matrix was more normal distributed. 

The calculation of the imbedded correlation showed how the error correlation matrix depends on the number of channels acquired with the spectrometers. It is of general knowledge that the signal acquisition is mostly an analogic process with a following data transmission and discretization to turn the signal digital and readable by smartphones and computers [41]. During this process, not only the sampling of the signal is carried out, but also the sampling of the noise. The spectrometers under investigation allow the acquisition with distinct parameters, including the possibility for a higher number of data points and interpolation options to return smooth signals. The technological development of this miniaturized systems and the evaluation of the different acquisition conditions are strictly related to frequencies and resolution of spectra that could influence the quality of the spectra in terms of noise. 

In addition, these parameters, as well as the data transmission process (through cable for the AvaSpec Mini-NIR and Bluetooth for the NeoSpectra Scanner), could be of importance because related to the time of data management and also influencing the associate estimated measurement error. 

The fact that different results are obtained by including different sources of variability in the calculation proved the importance of the definition of replicate, in accordance with the previous literature [5]. In addition, it could suggest that estimating the error relative to the different samples could be of interest if the result has to be used in further calculations to modify the algorithm [42] or determine uncertainty of models [17,43]. The results set the scene for further works that could include the investigation and evaluation of other methods for the estimation of covariance matrices of the measurement errors [44] and the comparison with covariance matrices constructed [45] by incorporating known sources of errors. Another interesting aspect could deal with the propagation of error when applying signal filters (as studied for the Kalman Filter [46]) or spectra preprocessing [47].

## 4. Concluding Remarks

In this research, several goals were achieved for the use of miniaturized spectrometers when analyzing a food sample of worldwide interest, sugar, in the lump and granulated form. Good evidence emerged from this study: performing the background at the beginning of each session or before every sample and doing the measurements in different analytical sessions influenced the entity of the measurement error but not the type of error substantially. This means that standard preprocessing methods employed in multivariate analysis can be suitable for preparing the data for multivariate modeling. Anyway, the influence of these variability sources on the measurement error depends on the sample and sensor characteristics. Thus, an evaluation of measurements error should be done before the optimization of every analytical method. As emerged from the literature but also from this work, miniaturized NIR spectrometers are powerful analytical tools with potentialities to individuate physical and chemical features in a huge variety of samples. However, they are still relatively unexplored, and this work lays the foundations for a new way of employing them, starting from the study of raw data measurements error.

## Figures and Tables

**Table 1:** Descriptive statistics of sugar spectra calculated on percentage reflectance.

	AvaSpec-Mini-NIR		NeoSpectra Scanner	
	Sugar Lump	Granulated Sugar	Sugar Lump	Granulated Sugar
Absolute standard deviation-mean	5.9	11.3	0.8	0.4
Relative standard deviation (%)-mean	25.5	37.9	5.3	3.05
RMS-mean	4.8	8.5	0.8	0.3
RMS-standard deviation	3.9	8.5	0.3	0.18
Signal/Noise Ratio-mean	4.0	2.7	19.7	42.4
Signal/Noise Ratio–standard deviation	0.6	0.4	5.4	19.8

**Table 2:** Percentage of variance accounted for in the original matrix of residuals as well as in the error covariance matrix by PCA models of increasing complexity for AvaSpec-Mini-NIR data.

	Sugar Lump		Granulated Sugar	
# of Principal Components	% Variance in Original Residuals (**Ê**)	% Variance in the Error Covariance Matrix (**Σ**)	% Variance in Original Residuals (**Ê**)	% Variance in the Error Covariance Matrix (**Σ**)
1	98.464	99.993	99.343	99.998
2	1.404	0.007	0.559	0.002
3	0.074	0.000	0.049	0.000
4	0.032	0.000	0.024	0.000
5	0.012	0.000	0.017	0.000

**Table 3:** Percentage of variance accounted for in the original matrix of residuals as well as in the error covariance matrix by PCA models of increasing complexity for NeoSpectra Scanner data.

	Sugar Lump		Granulated Sugar	
# of Principal Components	% Variance in Original Residuals (**Ê**)	% Variance in the Error Covariance Matrix (**Σ**)	% Variance in Original Residuals (**Ê**)	% Variance in the Error Covariance Matrix (**Σ**)
1	95.38	99.667	87.048	98.464
2	2.407	0.312	5.043	1.404
3	0.822	0.014	2.842	0.074
4	0.364	0.006	1.540	0.032
5	0.224	0.001	0.788	0.034

**Table 4:** K correlation indexes for the error matrix of NeoSpectra Scanner data. A indicate background acquired before each sample. B indicate background acquired before each session of analysis.

Imbedded Correlation	Sample	Background A			Background B		
		Session 1	Session 2	Session 3	Session 1	Session 2	Session 3
0.81	Sugar lump	0.85	0.83	0.91	0.87	0.89	0.89
	Granulated sugar	0.93	0.93	0.94	0.94	0.94	0.93

## Data Availability

Data available on request.

## Supplementary Materials

The following supporting information can be downloaded at: https://www.mdpi.com/article/10.3390/foods12030493/s1. Figure S1: Dataset obtained for each instrument; Figure S2: Loadings plot of the PCA model calculated for AvaSpec-Mini Error Covariance Matrix. (a) Sugar lump samples; (b) Granulated sugar samples; Figure S3: Diagonal of the Covariance Matrices calculated over all replicates spectra collect with AvaSpec-Mini-NIR on sugar lump samples (a) and granulated sugar (b) and with NeoSpectra Scanner on sugar lump samples (c) and granulated sugar (d); Figure S4: Loadings plot of the PCA model calculated for NeoSpectra Scanner Error Covariance Matrix. (a) Sugar lump samples; (b) Granulated sugar samples.

## References

[1] Chai J., Zhang K., Xue Y., Liu W., Chen T., Lu Y., Zhao G. (2020). Review of Mems Based Fourier Transform Spectrometers. Micromachines.

[2] Beć K.B., Grabska J., Huck C.W. (2021). Principles and Applications of Miniaturized Near-Infrared (NIR) Spectrometers. Chem. A Eur. J..

[3] Giussani B., Gorla G., Riu J. (2022). Analytical Chemistry Strategies in the Use of Miniaturised NIR Instruments: An Overview. Crit. Rev. Anal. Chem..

[4] Beć K.B., Grabska J., Huck C.W. (2022). Miniaturized NIR Spectroscopy in Food Analysis and Quality Control: Promises, Challenges, and Perspectives. Foods.

[5] Wentzell P.D. (2014). Measurement Errors in Multivariate Chemical Data. J. Braz. Chem. Soc..

[6] Bazar G., Kovacs Z., Tsenkova R. (2016). Evaluating Spectral Signals to Identify Spectral Error. PLoS ONE.

[7] Wentzell P.D., Lohnes M.T. (1999). Maximum Likelihood Principal Component Analysis with Correlated Measurement Errors: Theoretical and Practical Considerations. Chemom. Intell. Lab. Syst..

[8] Wentzell P.D., Wicks C.C., Braga J.W.B., Soares L.F., Pastore T.C.M., Coradin V.T.R. (2018). Implications of Measurement Error Structure on the Visualization of Multivariate Chemical Data: Hazards and Alternatives. Can. J. Chem..

[9] Karakach T.K., Wentzell P.D., Walter J.A. (2009). Characterization of the Measurement Error Structure in 1D 1 H NMR Data for Metabolomics Studies. Anal. Chim. Acta.

[10] Blanchet L., Réhault J., Ruckebusch C., Huvenne J.P., Tauler R., de Juan A. (2009). Chemometrics Description of Measurement Error Structure: Study of an Ultrafast Absorption Spectroscopy Experiment. Anal. Chim. Acta.

[11] Grenyer A., Erkoyuncu J.A., Zhao Y., Roy R. (2021). A Systematic Review of Multivariate Uncertainty Quantification for Engineering Systems. CIRP J. Manuf. Sci. Technol..

[12] Keller J.Y., Zasadzinski M., Darouach M. (1992). Analytical Estimator of Measurement Error Variances in Data Reconciliation. Comput. Chem. Eng..

[13] Martens H., Høy M., Wise B.M., Bro R., Brockhoff P.B. (2003). Pre-Whitening of Data by Covariance-Weighted Pre-Processing. J. Chemom..

[14] Zhu Y., Fearn T., Samuel D., Dhar A., Hameed O., Bown S.G., Lovat L.B. (2008). Error Removal by Orthogonal Subtraction (EROS): A Customised Pre-Treatment for Spectroscopic Data. J. Chemom..

[15] Gorla G., Taiana A., Boqué R., Bani P., Gachiuta O., Giussani B. (2022). Unravelling Error Sources in Miniaturized NIR Spectroscopic Measurements: The Case Study of Forages. Anal. Chim. Acta.

[16] Wentzell P.D., Brown S.D., Tauler R., Walczak B. (2009). 2.25—Other Topics in Soft-Modeling: Maximum Likelihood-Based Soft-Modeling Methods. Comprehensive Chemometrics.

[17] Allegrini F., Olivieri A.C. (2017). Recent Advances in Analytical Figures of Merit: Heteroscedasticity Strikes Back. Anal. Methods.

[18] Folch-Fortuny A., Vitale R., de Noord O.E., Ferrer A. (2017). Calibration Transfer between NIR Spectrometers: New Proposals and a Comparative Study. J. Chemom..

[19] Pereira L.S.A., Carneiro M.F., Botelho B.G., Sena M.M. (2016). Calibration Transfer from Powder Mixtures to Intact Tablets: A New Use in Pharmaceutical Analysis for a Known Tool. Talanta.

[20] Workman J.J. (2018). A Review of Calibration Transfer Practices and Instrument Differences in Spectroscopy. Appl. Spectrosc..

[21] Schoot M., Alewijn M., Weesepoel Y., Mueller-Maatsch J., Kapper C., Postma G., Buydens L., Jansen J. (2022). Predicting the Performance of Handheld Near-Infrared Photonic Sensors from a Master Benchtop Device. Anal. Chim. Acta.

[22] Eady M., Payne M., Changpim C., Jinnah M., Sortijas S., Jenkins D. (2022). Establishment of Instrument Operation Qualification and Routine Performance Qualification Procedures for Handheld Near-Infrared Spectrometers Used at Different Locations within a Laboratory Network. Spectrochim. Acta Part A Mol. Biomol. Spectrosc..

[23] Stinchcombe J.R., Simonsen A.K., Blows M.W. (2014). Estimating Uncertainty in Multivariate Responses to Selection. Evolution.

[24] Morad K.Y., Svrcek W., McKay I. (1999). A Robust Direct Approach for Calculating Measurement Error Covariance Matrix. Comput. Chem. Eng..

[25] Vasebi A., Hodouin D., Poulin É. (2013). The Importance of Uncertainty Covariance Tuning for Steady-State Data Reconciliation in Mineral and Metal Processing. IFAC.

[26] Sabahno H., Castagliola P., Amiri A. (2020). A Variable Parameters Multivariate Control Chart for Simultaneous Monitoring of the Process Mean and Variability with Measurement Errors. Qual. Reliab. Eng. Int..

[27] Leger M.N., Vega-Montoto L., Wentzell P.D. (2005). Methods for Systematic Investigation of Measurement Error Covariance Matrices. Chemom. Intell. Lab. Syst..

[28] Martínez M.L., Garrido-varo A., De Pedro E., Sánchez L. (1998). Effect of Sample Heterogeneity on near Infrared Meat Analysis: The Use of the RMS Statistic. J. Near Infrared Spectrosc..

[29] Adams M.J. (1995). Chemometrics in Analytical Spectroscopy.

[30] Zhang L., Xu H., Gu M. (2014). Use of Signal to Noise Ratio and Area Change Rate of Spectra to Evaluate the Visible/NIR Spectral System for Fruit Internal Quality Detection. J. Food Eng..

[31] Wentzell P.D., Giglio C., Kompany-Zareh M. (2021). Beyond Principal Components: A Critical Comparison of Factor Analysis Methods for Subspace Modelling in Chemistry. Anal. Methods.

[32] Matinrad F., Kompany-Zareh M., Omidikia N., Dadashi M. (2020). Systematic Investigation of the Measurement Error Structure in a Smartphone-Based Spectrophotometer. Anal. Chim. Acta.

[33] Todeschini R., Consonni V., Maiocchi A. (1999). The K Correlation Index: Theory Development and Its Application in Chemometrics. Chemom. Intell. Lab. Syst..

[34] Christian Huck Y.O., Huck C., Ozaki Y., Tsuchikawa S.B.E. (2014). Introduction and Principles. Near-Infrared Spectroscopy.

[35] Berntsson O., Danielsson L.G., Folestad S. (1998). Estimation of Effective Sample Size When Analysing Powders with Diffuse Reflectance Near-Infrared Spectrometry. Anal. Chim. Acta.

[36] Antila J., Tuohiniemi M., Rissanen A., Kantojärvi U., Lahti M., Viherkanto K., Kaarre M., Malinen J. (2000). MEMS-and MOEMS-Based Near-Infrared Spectrometers.

[37] Pasikatan M.C., Steele J.L., Spillman C.K., Haque E. (2001). Near Infrared Reflectance Spectroscopy for Online Particle Size Analysis of Powders and Ground Materials. J. Near Infrared Spectrosc..

[38] Szalay A., Antal I., Zsigmond Z., Marton S., Eros I., Regdon G., Pintye-Hódi K. (2005). Technical Note: Study on the Relationship between Particle Size and near Infrared Diffuse Reflectance Spectroscopic Data. Part. Part. Syst. Charact..

[39] Bro R., Smilde A. (2014). Principal component analysis. Anal. Methods.

[40] Bertinetto C.G., Schoot M., Dingemans M., Meeuwsen W., Buydens L.M.C., Jansen J.J. (2022). Influence of Measurement Procedure on the Use of a Handheld NIR Spectrophotometer. Food Res. Int..

[41] Cela R., Marcel Blanco V.C. (2007). Adquisicíon y Pre-Procesamiento de Señales Analíticas. Temas Avanzados de Quimiometría.

[42] Allegrini F., Braga J.W.B., Moreira A.C.O., Olivieri A.C. (2018). Error Covariance Penalized Regression: A Novel Multivariate Model Combining Penalized Regression with Multivariate Error Structure. Anal. Chim. Acta.

[43] Allegrini F., Wentzell P.D., Olivieri A.C. (2016). Generalized Error-Dependent Prediction Uncertainty in Multivariate Calibration. Anal. Chim. Acta.

[44] Feital T., Prata D.M., Pinto J.C. (2014). Comparison of Methods for Estimation of the Covariance Matrix of Measurement Errors. Can. J. Chem. Eng..

[45] Arteaga F., Ferrer A. (2013). Building Covariance Matrices with the Desired Structure. Chemom. Intell. Lab. Syst..

[46] Alfriend K.T., Vadali S.R., Gurfil P., How J.P., Breger L. (2010). Spacecraft Formation Flying.

[47] Roger J.-M., Boulet J.-C., Magida Zeaiter D.N.R. (2020). 3.01—Pre-Processing Methods. Comprehensive Chemometrics.

