# Supplementary material for: On the Importance of Investigating Data Structure in Miniaturized NIR Spectroscopy Measurements of Food: The Case Study of Sugar
Source: Foods. 2023 Jan 20;12(3):493. doi: 10.3390/foods12030493 (PMC9914682; doi:10.3390/foods12030493)

Supplementary Material

Supplementary Figure S1. Dataset obtained for each instrument.

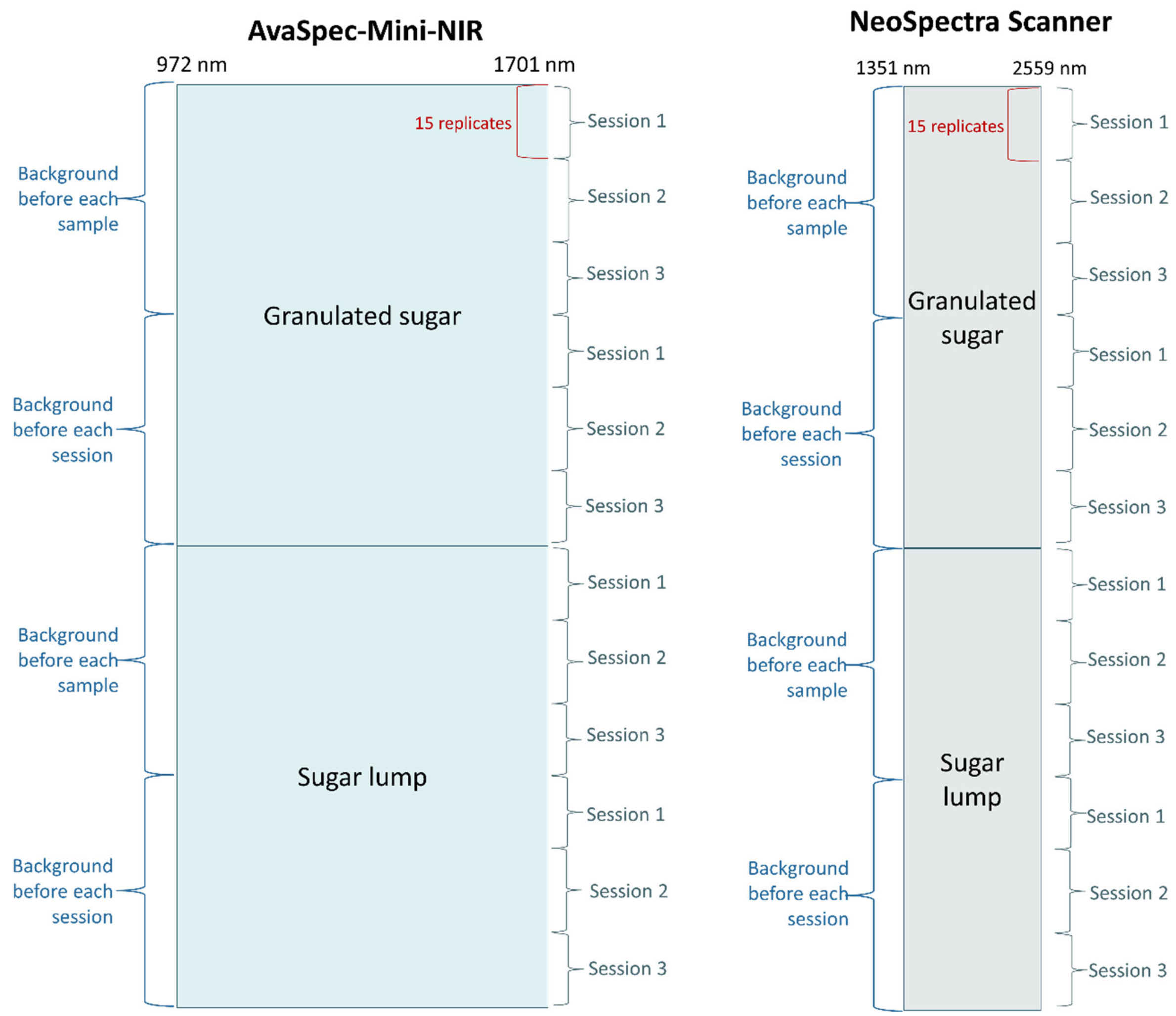

Supplementary Figure S2. Loadings plot of the PCA model calculated for AvaSpec-Mini Error Covariance Matrix. a) Sugar lump samples; b) Granulated sugar samples.

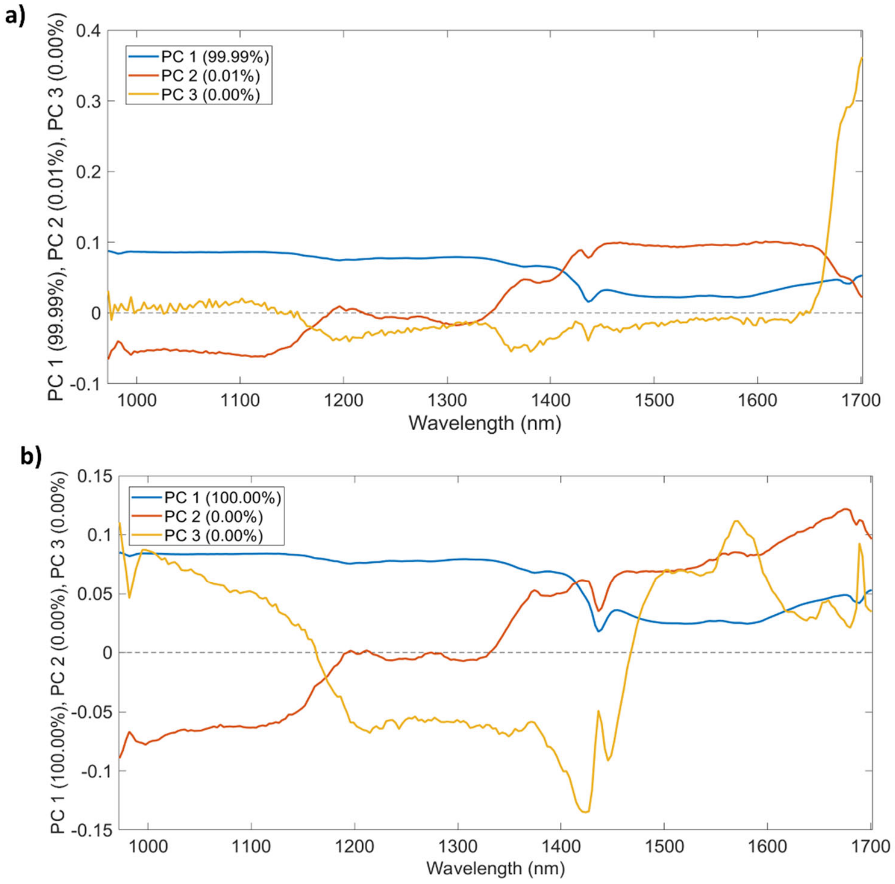

Supplementary Figure S3. Diagonal of the Covariance Matrices calculated over all replicates spectra collect with AvaSpec-Mini-NIR on sugar lump samples (a) and granulated sugar (b) and with NeoSpectra Scanner on sugar lump samples (c) and granulated sugar (d).

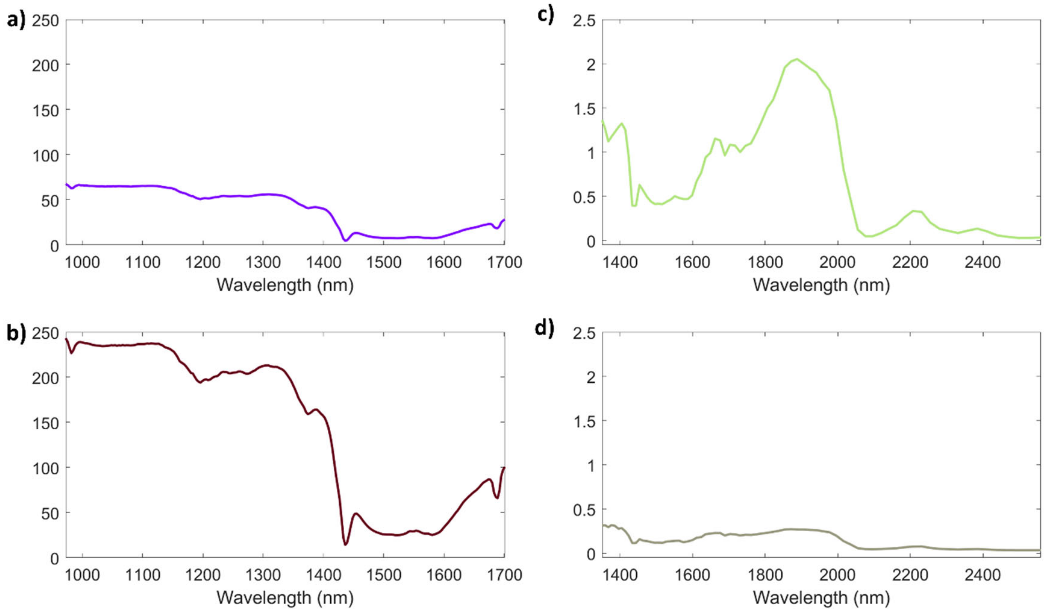

Supplementary Figure S4. Loadings plot of the PCA model calculated for NeoSpectra Scanner Error Covariance Matrix. a) Sugar lump samples; b) Granulated sugar samples.

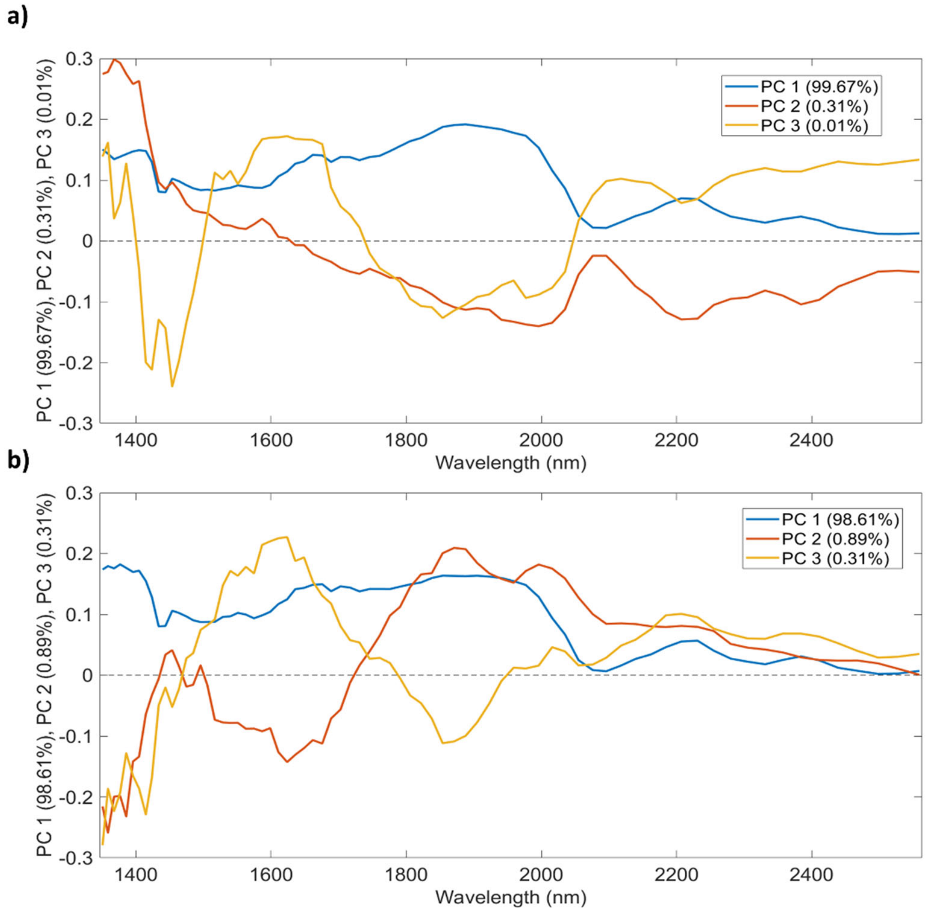

Supplement: Supplementary file 1 [file foods-12-00493-s001.zip › foods-2141202-supplementary.pdf]
